# Acute intraparenchymal cerebral haemorrhage in an Iberian golden eagle – a case report

**DOI:** 10.1186/s12917-018-1379-2

**Published:** 2018-02-27

**Authors:** Cláudia S. Baptista, Carla Monteiro, Hélder Fernandes, Ana Canadas, Luísa Guardão, Joana C. Santos

**Affiliations:** 1 Department of Veterinary Clinics, UPVet, Institute of Biomedical Sciences Abel Salazar, University of Porto (ICBAS-UP), Rua Jorge Viterbo Ferreira 228, 4050-313 Porto, Portugal; 2 Instituto de Ciências e Tecnologias Agrárias e Agro-Alimentares da Universidade do Porto – Centro de Estudos de Ciência Animal (ICETA-CECA), Rua D. Manuel II, Apartado 55412, 4051-401 Porto, Portugal; 3 Santo Inácio Zoo, Rua 5 de Outubro 4503, 4430-809 Avintes, Portugal; 4 Clínica Veterinária das Taipas, Rua António Barros 266, Caldelas, 4805-087 Caldas das Taipas, Portugal; 5 Department of Molecular Pathology and Immunology, Veterinary Pathology Laboratory, Institute of Biomedical Sciences Abel Salazar, University of Porto (ICBAS-UP), Rua Jorge Viterbo Ferreira 228, 4050-313 Porto, Portugal

**Keywords:** Iberian golden eagle, Acute cerebral haemorrhage, Computed tomography, CT head anatomy

## Abstract

**Background:**

In birds there are reports of intracranial lesions but not of the clinical, computed tomographic and histopathologic features of acute intraparenchymal cerebral haemorrhage in Iberian golden eagle.

**Case presentation:**

The following report describes a case of a 30-year-old Iberian golden eagle *(Aquila chrysaetos homeyeri)* with no history of trauma, presented with acute opisthotonus, left head tilt and circling, anisocoria, positional nystagmus, and ataxia. The main differential diagnosis were hypovitaminosis B or E and intracranial disease due to trauma, infection, toxins or masses. A computed tomography (CT) of the head was performed with an 8-slices scanner and evidenced a hyperdense (63–65 HU) non-enhancing homogeneous well delineated round area in the midbrain, with 6 mm in its highest diameter. The attenuation values and the non-enhancing nature of the lesion strongly suggested the diagnosis of acute intraparenchymal haemorrhage, which was histologically confirmed after necropsy.

**Conclusions:**

In birds with a central neurological dysfunction, the diagnosis of acute brain haemorrhage should be considered when the CT evidences a non-enhancing, homogeneous, well circumscribed hyperattenuated round area.

## Background

The Iberian golden eagle (*Aquila chrysaetos homeyeri*) is a subspecies of the golden eagle that inhabits the Iberian Peninsula. It has slight variations than in the form *Aquila chrysaetos chrysaetos* of northern Europe, namely smaller size and darker coloration, with less marked shades. The golden eagle lives preferentially in mountainous areas, but it is not uncommon in places of low altitude or depopulated [1].

In dogs and cats, the most common cause of brain hemorrhage is trauma [2], but infectious diseases, toxins, intracranial neoplasia, von Willebrand factor deficiency and other coagulopathies, parasitic migration, cerebral vascular malformation, peripartum asfixia and idiopathic etiology may also lead to excessive bleeding [3, 4]. The hemorrhage can be extra-axial (epidural, subdural, subarachnoid / intraventricular) and/or intra-axial (cortical contusion, intraparenchymal hematoma and traumatic axonal or shear injury) [3, 5]. In companion animals, common causes of acute head trauma include high impact automobile collisions, violent blunt force and missile injuries (e.g. gunshot wounds), animal bites and lower impact injury from falls and collisions [6, 7]. For diagnosis of acute traumatic brain injury, computed tomography (CT) is the modality of choice as it quickly and accurately identifies skull fractures and intracranial hemorrhage [7].

In birds, head trauma is usually due to crashes into trees, windows, power lines or cars and can be a cause of fractures of sclera bones, sub−/luxation of lens [8] and central neurologic signs. Computed tomographic studies of the central nervous system and eyes, such as acute cases of hemorrhage, trauma, malformation and neoplasia, may be of similar diagnostic value in birds as in dogs and cats [9].

To the authors knowledge there are no reports describing the CT characteristics of acute intraparenchymal cerebral haemorrhage in the golden eagle, or even in raptors. Accordingly, the aim of this case report is to characterize the clinical signs and the computed tomographic features of a histologically confirmed midbrain haemorrhage in an Iberian golden eagle.

## Case presentation

A 30-year-old Iberian golden eagle (*Aquila chrysaetos homeyeri*) from a zoo in the region of Porto-Portugal, was presented with disorientation, acute opisthotonus, left head tilt and circling, anisocoria, positional nystagmus, and inability to fly and stand. There was no known history of trauma and no external lesions were visible. The animal was placed in a warm, oxygenated incubator with intravenous fluid therapy (ringer lactate 120 ml/kg/daily in bolus), meloxicam (0.2 mg/kg/daily subcutaneous injection), itraconazol (8 mg/kg, twice daily, oral admnistration) and enrofloxacin (10 mg/kg/daily, intramuscular injection). The main differential diagnosis considered were hypovitaminosis B or E and intracranial disease due to trauma, infection (bacterial, fungal, viral), toxins or cerebral mass (granuloma, hematoma, abscess, neoplasia).

**Fig. 1:**
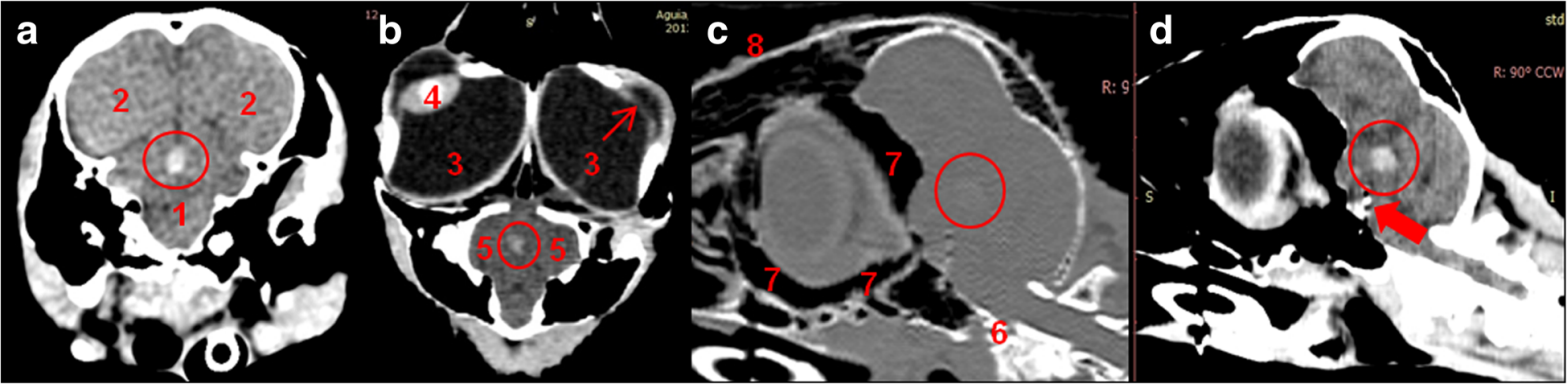
In vivo images of the head obtained with helical computed tomography. **a** Axial image. **b** Dorsal reconstruction. **c** Sagittal reconstruction with bone algorithm. **d** Sagittal reconstruction with soft tissue algorithm. **a** and **b** Pre-contrast images; **c** and **d** Post-contrast images. There is a hyperdense nonenhancing homogeneous well delineated round area with a density of 65 HU and 6 mm in its highest diameter (6 × 4.4 × 5.4 mm) in the midbrain (circle). Such lesion is suggestive of acute intraparenchymal hemorrhage. 1. Midbrain; 2. Telencephalon; 3. Vitreous; 4. Lens; 5. Optic lobes; 6. Preorbital, infraorbital and postorbital parts of the paranasal sinus; 7. Paranasal sinus; 8. Spongi bone; Thin arrow: cataract; Gross arrow: note the contrast uptake at the pituitary region

Hematologic parameters were normal and, when considered clinically stable (next day following presentation), the golden eagle was transported to the Small Animal Veterinary Hospital of the University of Porto (UPVet) in order to perform a computed tomography of the head with an 8-slices scanner (Lightspeed, GE Healthcare Life Sciences, Carnaxide, Portugal). The animal was anesthetized with isofluorane, positioned in sternal recumbency and taped to the CT table. Axial images were acquired with a helical scanning mode at 200 mA, 120 Kv, 0.6 mm slice thickness and using soft tissue and bone reconstruction algorithms, before and after intravenous injection of iodinated contrast medium (250 mg I/Kg) administered into the basilic vein. Dorsal and sagittal reconstructions were also obtained from transverse images. CT scans showed a cataract in the left eye and a hyperattenuated non-enhancing homogeneous well delineated round area, ventral to the third ventricle, with a density of 63 to 65 HU and 6 mm in its highest diameter (6 × 4.4 × 5.4 mm). This lesion did not produce deviation of the *falx cerebri* and was confined to the midbrain with no evidence of injury in the ventricular system (Fig. 1). Such lesion was suggestive of acute intraparenchymal haemorrhage/hematoma but additional differential diagnosis such as granuloma or neoplasia could also be considered.

**Fig. 2:**
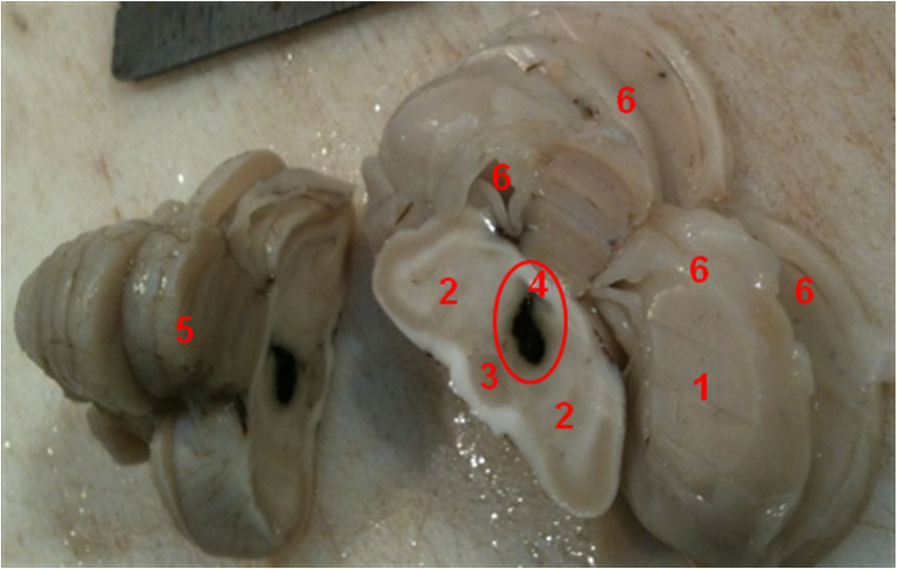
Macroscopic view of the brain. We can observe a well limited black area, after fixation with 10% formalin, compatible with a recent intraparenchymal hemorrhage (circumference). 1. Cerebrum; 2. Midbrain, optic lobes; 3. Pituitary gland; 4. Third ventricle; 5. Cerebellum; 6. Lateral ventricles

**Fig. 3:**
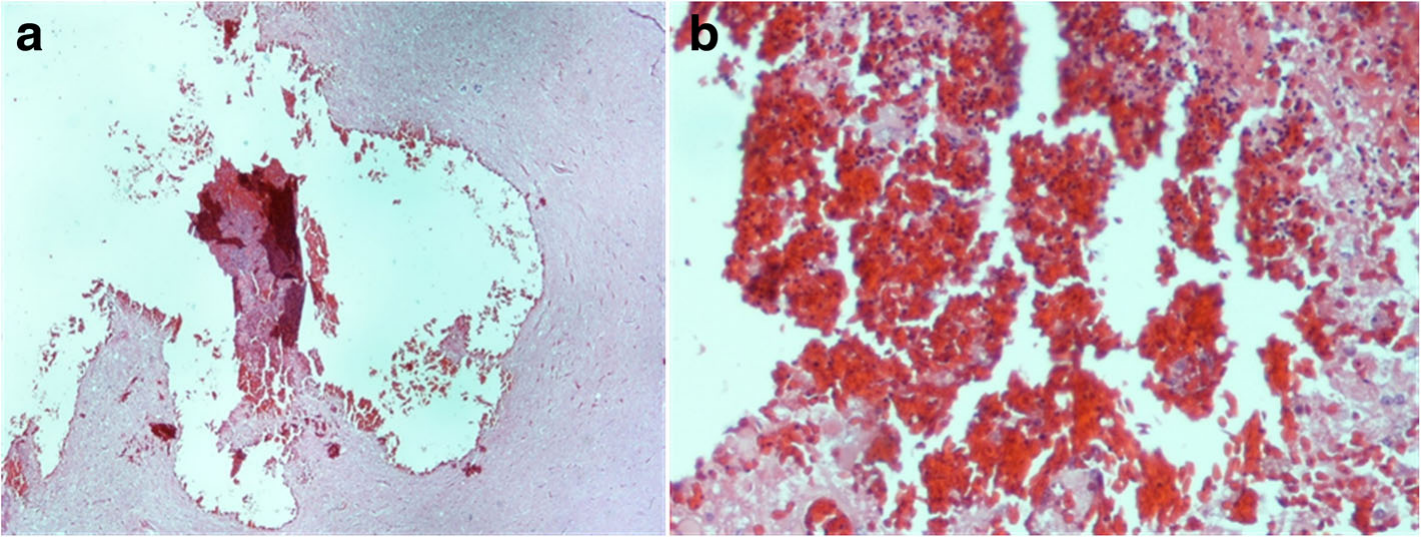
**a** Histopathology of the intraparenchymal hemorrhage (HE20x): the red area corresponds to the recent hemorrhage. **b** Detail of A (HE400x): extravasated well-preserved red blood-cells (RBC) due to the hemorrhage, without any inflammation

One day after the CT scan the eagle was euthanized for humanitarian reasons and the brain was removed and dissected after fixation with 10% formalin. Post-mortem examination revealed the presence of a focal area of black tissues in the parenchyma of the midbrain, consistent with haemorrhage (Fig. 2). Brain slices were processed routinely for paraffin embedding and stained with haematoxylin and eosin (HE). Histological examination confirmed the focal intraparenchymal recent (or acute) haemorrhage, characterized by extravasated well-preserved red blood cells (RBC) without inflammatory cells population (Fig. 3).

## Discussion and conclusions

In birds, besides trauma, other differential diagnoses for central nervous system dysfunction include infectious diseases (e.g. viral, parasitic, bacteria and fungus), nutritional imbalances, toxins (e.g., heavy metals, organophosphates, pyrethroids, drugs and plants), neoplasia, congenital abnormalities and cardiovascular, hepatic and renal disorders [10].

The assessment of the neurological bird should include a thorough history, a detailed external physical examination, haematology and serum biochemistry, radiography, ultrasonography and endoscopy. Additional diagnostic tests such as heavy metal analysis, serology/PCR for infectious diseases and cerebral spinal fluid analysis may be required in some clinical cases [10]. Although there was no evidence of any external injury, the shelter of this eagle was made of wood and net, with natural soil substrate, so trauma can also be considered a probable cause of intracranial disease, here evidenced by the clinical signs presented (acute opisthotonus, left head tilt and circling, anisocoria and ataxia).

Diagnostic imaging procedures such as radiology, computed tomography and magnetic resonance imaging (MRI) are extremely useful to evaluate the severity of the head injury, namely to identify fractures and the presence of bleeding. These diagnostic tools also help in determining the presence of foreign bodies, congenital abnormalities and masses such as granuloma, hematoma, abscess or neoplasia [11].

As previously mentioned, in this clinical case there was strong evidence of a central nervous system disorder so, considering that clinical neurological signs are rarely pathognomonic [10], a multiplanar imaging technique was the first choice procedure to rule out the differential diagnosis, namely computed tomography. In fact, CT can be more useful than MRI to characterize bone lesions [6], which are most likely to occur in trauma patients, and is an excellent method for the detection of acute brain hemorrhage [6, 12] due to the high content of globin and fibrin resulting in a hyperattenuated area [12]. Additionally, CT images are acquired more quickly than MR images and patients may be more closely monitored with standard monitoring systems [6]. However, MRI has better diagnostic sensitivity for nonhemorrhagic contusions and shear-strain injuries [5] and is preferred when clinical signs are not explained by CT findings or in patients with subacute to chronic brain trauma [11].

Although there are no publications describing the computed tomographic characteristics of acute intraparenchymal haemorrhage in raptors, CT has been used successfully to locate intracranial lesions in birds with a reported sensitivity of 80% [13, 14]. MRI was valuable for detecting brain lesions and defining their three-dimensional distribution and extent in birds with lead poisoning, which is an important cause of central nervous system damage in bald eagles (*Haliaeetus leucocephalus*). In the previous study, one animal presented a multifocal acute intraparenchymal haemorrhage [15]. Additionally, there is one publication describing the MRI features of a recent hemorrhage in a Grey parrot (*Psittacus erithacus*), but no abnormalities were observed in the CT scans [16].

The CT image of the lesion described in this case report evidenced attenuation values around 63–65 HU which are within the limits recognized for acute haemorrhage (60–80 HU) [12, 17]. Additionally, the well defined limits and its non-enhancing nature, due to the poor blood perfusion of the acute phase of the haemorraghic event, strongly suggested the diagnosis of an acute haemorrhage [12] localized in the midbrain. Lesions in the midbrain may produce depression, disorders of ocular movement, constricted pupils, poor or absent pupillary light reflexes, gait deficits, and contralateral or ipsilateral postural deficits [2], which are compatible with the clinical signs here reported.

In humans, dogs and cats, considerable controversy exists concerning what constitutes appropriate therapy for severe brain-injured patients. However, it is commonly accepted that is essential to alleviate brain swelling and prevent damage to vital brain-steam structures. The ultimate goal is to return the patient to the role in society occupied prior to the injury [6]. In birds of prey the inability to fly is incompatible with quality of life, so the animal was humanely euthanized according to the veterinary best practices.

This case report contributes for the imaging characterization of intracranial disease and normal CT anatomy in wild animals, namely in *Aquila chrysaetos homeyeri*. In raptors with a central neurological dysfunction, the diagnosis of acute intraparenchymal haemorrhage should be considered when the CT evidences a non-enhancing, homogeneous, well circumscribed hyperattenuated round area.

## Abbreviations

CT: Computed tomography
HE: Haematoxylin and eosin stain
HU: Hounsfield unit
MRI: Magnetic resonance imaging
PCR: Polymerase chain reaction
RBC: Red blood cells
